# Sex-Specific Effect of the Dietary Protein to Carbohydrate Ratio on Personality in the Dubia Cockroach

**DOI:** 10.3390/insects13020133

**Published:** 2022-01-27

**Authors:** Sofia Bouchebti, Fernando Cortés-Fossati, Ángela Vales Estepa, Maria Plaza Lozano, Daniel S. Calovi, Sara Arganda

**Affiliations:** 1 Departamento de Biología y Geología, Física y Química Inorgánica, Área de Biodiversidad y Conservación, Universidad Rey Juan Carlos, 28933 Madrid, Spain; fernando.cfossati@urjc.es (F.C.-F.); a.vales.2016@alumnos.urjc.es (Á.V.E.); m.plazal.2017@alumnos.urjc.es (M.P.L.); sara.arganda@urjc.es (S.A.); 2 Department of Collective Behaviour, Max Planck Institute for Ornithology, 78464 Konstanz, Germany; daniel.calovi@gmail.com; 3 Centre for the Advanced Study of Collective Behaviour, University of Konstanz, 78464 Konstanz, Germany

**Keywords:** nutrition, macronutrient proportion, exploratory behavior, *Blaptica dubia*, survival

## Abstract

**Simple Summary:**

Animal personality is modulated by genetic and environmental factors. To explore the modulatory effect of nutrition on personality, we investigated whether diets varying in their relative content of proteins and carbohydrates might modulate the behavior of the Dubia cockroach. Over a period of eight weeks, we fed adult cockroaches, both males and females, five different diets, and we measured diet consumption, survival, and personality traits by recording their exploratory and mobility behaviors. After eight weeks, females gained more body mass and had higher survival than males. We found that females preferred carbohydrate-rich diets and avoided ingesting too many proteins by consuming less food on high-protein diets. The diet had no effect on their personality. However, males showed a bolder personality when fed with high-protein diets while consuming the same amount of food, regardless of the protein content in the diet. These sex differences could be beneficial for the species in stressful nutritional environments, allowing males to discover new food resources while ovoviviparous females could spend more time protected in shelters.

**Abstract:**

Animal personality, defined by behavioral variations among individuals consistent over contexts or time, is shaped by genetic and environmental factors. Among these factors, nutrition can play an important role. The Geometric Framework of Nutrition has promoted a better understanding of the role of the macronutrient proportion in animal development, survival, reproduction, and behavior, and can help to disentangle its modulatory effect on animal personality. In this study, we investigated the effects of protein to carbohydrate (P:C) ratio in the personality of the cockroach *Blaptica dubia*. Newly emerged adults were fed over a period of eight weeks on five different diets varying in their P:C ratio and their diet consumption, mass variation, survival, exploratory behavior, and mobility were assessed. We found that females, unlike males, were able to regulate their nutrient intake and preferred carbohydrate-rich diets. Females also gained more body mass and lived longer compared to males. In addition, their behavior and mobility were not affected by the diet. In males, however, high-protein diets induced a bolder personality. We suggest that the sex-specific effects observed on both survival and behavior are related to the nutrient intake regulation capacity and might improve the species’ fitness in adverse nutritional conditions.

## 1. Introduction

Animal personality is defined by inter-individual behavioral variations consistent over contexts or time [1]. Although the age of an individual can intensify some behavioral traits [2], personality is generally constant across different life stages [3,4]. Most of the studies focused on personality address variations in activity, boldness, or aggressiveness [5]. These personality traits affect individuals’ fitness in many species [6]. For instance, aggressive male cockroaches have a higher probability to mate [7] and, in many species, bolder individuals or individuals presenting a higher activity level exhibit a more important growth rate as they spend more time exploring their environment and find more food resources [8]. However, the higher tendency to explore also increases the risk of predation [8,9,10,11,12,13,14]. Hence, bolder animals usually present a shorter lifespan [15]. For animals living in groups, more efficient decision-making processes can be achieved by different individuals’ personalities, improving the fitness of the whole group [16]. Personality is also observed at a collective level [17]. Thus, in the same manner as for solitary animals, colonies of social insects, such as bees or ants, exhibit collective personalities also affecting their fitness [18,19,20].

Personalities are shaped by the combined effects of genetic and environmental factors [21]. Among environmental factors, temperature [22], parasitism [23], social environment [24,25], or even urbanization [26] have been shown to induce different personalities. Nutrition can also play a determinant role in shaping personalities [27]. For instance, in the mustard leaf beetle *Phaedon cochleariae*, individuals raised on a low-quality diet had bolder personalities once adults [28], while in the Madagascar hissing cockroach *Gromphadorhina portentosa,* nymphs raised on low-quality diets exhibited a decrease of exploration activity in adult life [29].

The emerging field of nutritional geometry can be used to untangle the role of specific nutrients in shaping personality [27,30]. The nutritional geometry framework constitutes a set of methodological and interpretative tools conceived to study how nutrients and their interactions might affect the phenotypes and behaviors of organisms, and has been used, for example, to study how individuals regulate their intake of nutrients to maximize their fitness [31]. Numerous studies using this framework approach have shown that unbalanced diets of proteins and carbohydrates can affect life-history traits and lead to trade off fitness traits such as lifespan, reproduction, and immunity [32]. However, to the best of our knowledge, only one study has used this approach to investigate the role of nutrition in shaping personalities, using the southern field cricket *Gryllus bimaculatus* as a model [33]. The authors found a sex-specific effect of the diet, with male individuals raised on high protein ratio diets exhibiting a more aggressive personality [33].

Here, we aim to investigate the role of macronutrients (proteins and carbohydrates) in shaping the personality of the Dubia cockroach (*Blaptica dubia)*. Cockroaches are an ideal model to study personality as they exhibit various individual [2,7,29] and collective [17] personalities. They are considered extreme generalists and are able to adapt to any nutritional deficiency [34,35], thus allowing us to explore the effects of the widest ranges of unbalanced diets.

Newly emerged adults (males and females) were fed on fixed diets varying in protein to carbohydrate ratio (P:C ratio) or a choice diet allowing them to balance their intake of P:C ratio, and diet consumption, survival, and personality traits (boldness and exploration) were assessed over a period of eight weeks.

## 2. Material and Methods 

### 2.1. Experimental Individuals

Individuals of *Blaptica dubia* were obtained from a breeding colony originally purchased in two different pet stores (Harkito Reptile, Madrid, Spain, harkitoreptile.com, accessed on 26 January 2022; and Animal Center Valencia S.L., lagrillera.com, accessed on 26 January 2022). The cockroaches were maintained in a plastic box (24 × 35 × 14.5 cm) under our standard laboratory conditions (25 °C, 55% humidity, 12:12 light:dark photoperiod) with ad libitum access to shelters (egg cartons and cardboard cylinders), food (cat dry chow and fruits), and water.

To obtain experimental subjects of the same stage of development, all the final instar nymphs (approximately 100 nymphs) were collected and isolated in a plastic box in the same conditions as previously described. Every day, newly emerged adults were collected (i.e., when males and females presented wings and reduced wings, respectively), placed in an individual cage (13.5 × 5.5 × 3.5 cm), and randomly allocated to an experimental diet (one of the four fixed diets or the choice diet). Each individual cage contained one or two feeders (Petri dishes, 320 mm ø) depending on the experimental group, a 1.5 mL Eppendorf tube filled with water and clogged with cotton, and a shelter (aluminum foil).

To assess food consumption, feeders were weighed and renewed every week. To assess the evaporation rate, control feeders containing the diets were weighed in the same manner and placed in boxes without cockroaches. The cockroaches were weighed at the beginning and at the end of the experiment (or at their death). Dead individuals were counted every weekday.

### 2.2. Diets

Four different artificial diets were prepared, defined by their P:C ratio: 1:0, 0:1, 2:1, and 2:1, using sucrose and a mix of amino acids as carbohydrate and protein sources, respectively. All diets contained cholesterol, Wesson’s salts, and Vanderzant vitamin mixture for insects (Table 1, diets modified from [36]) (Sigma-Aldrich, St. Louis, MO, USA). To obtain a homogenous paste, 0.20 mL of water was added for 1 mg of each diet. Individuals were randomly allocated to one of the four fixed diets (0:1 diet, n = 11 females and n = 13 males; 1:2 diet, n = 10 females and n = 11 males; 2:1 diet, n = 10 females and n= 16 males; and 1:0 diet, n = 11 females and n = 21 males) or to the choice experiment where individuals had access simultaneously to the 1:0 ratio and 0:1 ratio diets (n = 10 females and n = 13 males).

### 2.3. Behavioral Protocol

The behaviors of each individual were tested during their active period (dark photoperiod phase) every two weeks for eight weeks. All the experiments were performed under red light (which is not perceived by cockroaches [37]). Each cockroach was placed by hand in a shelter (9 × 9 × 4 cm) in the center of the arena (50 × 35 × 8 cm). After a three minute habituation period, the door of the shelter was opened and the behavior of the individual was video recorded for 20 min (Sony Handycam 4K FDR-AX33). The latency of the first antennae out of the shelter and the latency of the individual exiting the shelter (six legs out the shelter) were noted and used to calculate the decision time to leave the shelter. Bolder individuals were defined by a shorter decision time to leave the shelter.

The arena and the shelter were cleaned with a solution of 96% ethanol before each trial. 

### 2.4. Trajectories Analyses

Trajectories performed by each individual in the exploration arena were analyzed by an automatic visual monitoring consisting of extracting the background and detecting the remaining elements that differed from the background above a brightness threshold (blob detection). Given the simplicity of the tracking problem (single individual in a static environment with constant lighting), the tracking software was made in-house using MATLAB 2021b. To calculate the position of the cockroach, we used the centroid of the detected blob which had an area higher than a custom threshold, smaller than the full size of the individual. This was chosen due to the instances where the individuals tried to climb the enclosure, and in doing so, drastically reduced their visual profile. This also means that tracking started once a sufficiently large portion of the cockroach had left the shelter. Having obtained the position of the cockroaches in a given frame, the actual dimensions of the experimental area and the position of its corners (manually marked for each video with the help of the Fiji program [38]) were used to calculate a geometric transformation that mapped the coordinates of the pixels, even in perspective, to the metric coordinates of the confinement. Finally, with the positions of the individuals in meters, the metrics of interest were calculated: speed, traveled distance, and exploration ratio. Speed was calculated using a time window of 2 s. The traveled distance of each cockroach was obtained by integrating speed over time (speed × dt); note that by using the speed (and hence a 2 s time window) to calculate the traveled distance, we minimized the effects of noise on estimating the centroids of each blob adding to the overall traveled distance. We also normalized both speed and traveled distance using body-length units. The body size of each cockroach was estimated using the major axis of the corresponding blob. The averages of each variable per cockroach were calculated. Subsequently, the speed and traveled distance were normalized by the average body length of each cockroach to avoid a bias in the data, since longer individuals travel greater distances at greater speeds than those of a smaller size. Finally, we created a metric, the exploration ratio, that accounts for how much of the available area each cockroach explored in a single experiment (with bins of one square centimeter), i.e., a cockroach that barely left the shelter would have an exploration ratio near zero, while one that had visited nearly every square centimeter of the area would tend to one.

### 2.5. Statistical Analyses 

Food consumption was compared between diets per sex and per week, separately, and between weeks per diet and per sex using Kruskal–Wallace rank-sum tests, followed by Dunn’s tests of multiple comparisons. Food consumption was also compared between sexes per week with Wilcoxon rank-sum tests.

For the choice experiment, the protein and carbohydrate intakes accumulated were compared between males and females with Wilcoxon tests.

We used a linear model to compare the body mass differences between the end and the beginning of the experiment. The model included diet, sex, and their interaction.

Survival analysis was performed using a Cox proportional hazards regression model considering censored data. The model included diet, sex, and their interaction.

The decision time to leave the shelter was calculated by subtracting the latency to leave the shelter (six legs outside the shelter) to the latency of the first antenna out (i.e., when individuals noticed the open door). The decision time to leave the shelter was then compared between diets per sex and per week, separately, and between weeks per diet and per sex using Kruskal–Wallace rank-sum tests, followed by Dunn’s tests of multiple comparisons. Wilcoxon tests were used to compare the decision time to leave the shelter between males and females for each week.

The metrics calculated by the video tracking of trajectories (speed in body lengths per second, traveled distance in body lengths, and exploration ratio) were analyzed using linear mixed models (R function *lmer*) and pairwise comparisons (R function *emmeans*). Linear models for speed and traveled distance had diet, sex, week, and their interactions as fixed variables and individual id as a random factor (on the intercept and on weeks). Exploration ratio, not normalized by body length, had body length as an additional fixed factor. Linear models were simplified by backward selection.

All analyses were done on R 4.0.3 [39], and significance was determined using α = 0.05.

## 3. Results

### 3.1. Females Regulate Their Nutrient Intake, Consume More Carbohydrates and Gain More Body Mass

**Figure 1:**
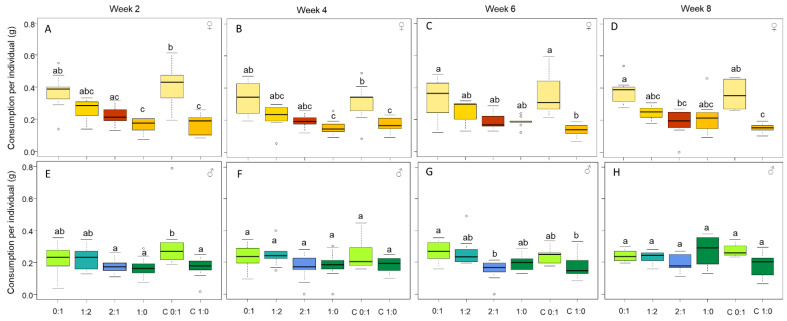
Food consumption (g) per week for females (week 2 (**A**), week 4 (**B**), week 6 (**C**), and week 8: (**D**)) and males (week 2 (**E**), week 4 (**F**), week 6 (**G**), and week 8: (**H**)). Individuals fed on the four different fixed diets (0:1, 1:2, 2:1, and 1:0 P:C) and on the choice diet (C0:1 and C1:0 P:C). The boxes represent the first and third quartiles and the median. The whiskers represent the maximum and minimum values. The circles represent the outliers. Different letters indicate a pairwise comparison with *p* ≤ 0.05.

The diet influenced the weekly quantity of food that the female cockroaches consumed throughout the experiment (Figure 1A, week 2: H = 36.68, DF = 5, *p* < 0.0001; Figure 1B, week 4: H = 27.31, DF = 5, *p* < 0.0001; Figure 1C, week 6: H = 22.26, DF = 5, *p* < 0.001; Figure 1D, week 8: H = 24.02, DF = 5, *p* < 0.001). Females regulated their intake of protein by consuming smaller quantities of food with high P:C ratio diets. Males, on the other hand, generally consumed the same quantity of food, regardless of the diet (Figure 1E, week 2: H = 21.83, DF = 5, *p* < 0.001; Figure 1F, week 4: H = 9.25, DF = 5, *p* = 0.099; Figure 1G, week 6: H = 19.89, DF = 5, *p* < 0.05; Figure 1H, week 8: H = 9.36, DF = 5, *p* = 0.095).

The consumption of each diet did not change over the weeks for either females or males (Figure 1, females: diet 0:1 P:C, H = 0.43, DF = 3, *p* = 0.933; diet 1:2 P:C, H = 2.74, DF = 3, *p* = 0.432; diet 2:1 P:C, H = 2.47, DF = 3, *p* = 0.480; diet 1:0 P:C, H = 3.74, DF = 3, *p* = 0.290; choice diet 0:1 (C 0:1), H = 7.51, DF = 3, *p* = 0.377; and choice diet 1:0 (C 1:0), H = 13.46, DF = 3, *p* = 0.061; males: diet 0:1 P:C, H = 1.87, DF = 3, *p* = 0.599; diet 1:2 P:C, H = 0.60, DF = 3, *p* = 0.895; diet 2:1 P:C, H = 0.94, DF = 3, *p* = 0.813; diet 1:0 P:C, H = 5.83, DF = 3, *p* = 0.120; choice diet 0:1 (C 0:1), H = 5.23, DF = 3, *p* = 0.631; and choice diet 1:0 (C 1:0), H = 2.62, DF = 3, *p* = 0.917).

Additionally, females consumed more than males at the beginning of the experiment (week 2, z = 2274.00, *p* = 0.005; week 4, z = 1452.50, *p* = 0.677; week 6, z = 903.00, *p* = 0.637; and week 8, z= 592.50, *p* = 0.537).

**Figure 2:**
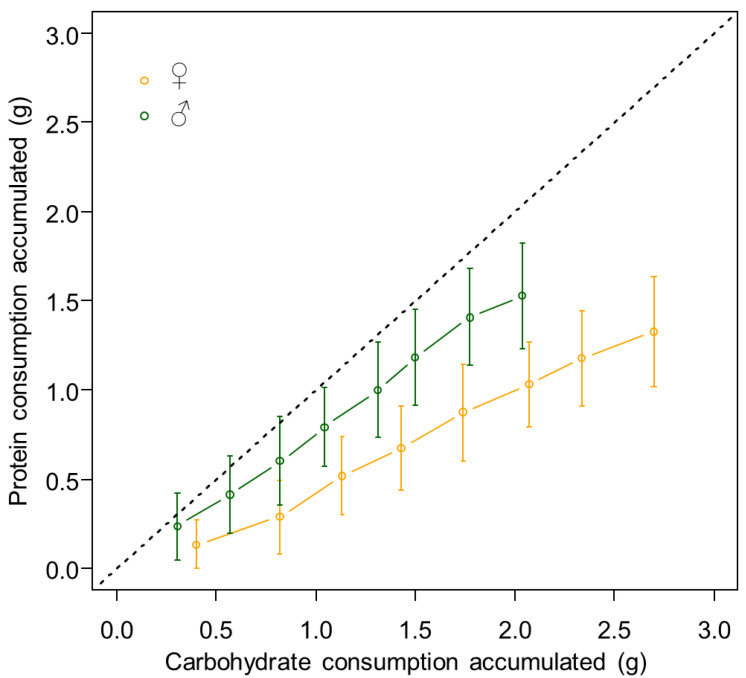
Mean consumption of protein and carbohydrate accumulated (g) during the 8 weeks when individuals had access to the choice diet. The error bars represent the standard deviations. The dotted black line represents the expected intake trajectory if feeding had occurred randomly between the two diets.

In the choice experiment, where individuals could balance their intake of protein and carbohydrate, females and males consumed respectively the same quantity of 0:1 and 1:0 diets as they did when restricted to the fixed diets 0:1 and 1:0 (Figure 1). The consumption of protein did not differ between males and females (Figure 2, z = 2317.50, *p* = 0.085); however, females consumed more carbohydrates (Figure 2, z = 3973.50, *p* < 0.0001).

**Figure 3:**
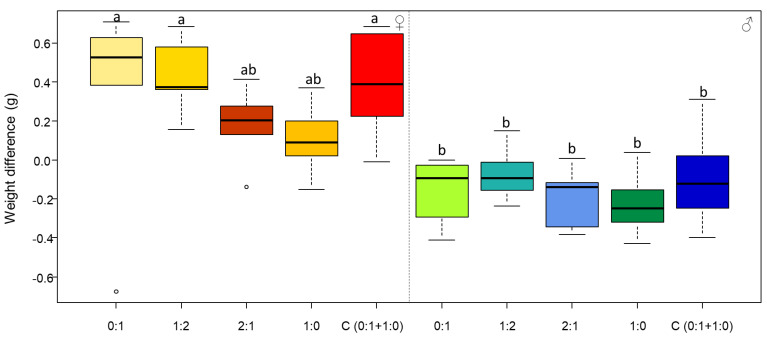
Body mass gain (body mass difference between the end and the beginning of the experiment) per diet (fixed diets: 0:1, 1:2, 2:1, and 1:0 P:C; and choice diet: C (0:1 + 1:0)) for female (♀) and male (♂) individuals. The boxes represent the first and third quartiles and the median. The whiskers represent the maximum and minimum values. The circles represent the outliers. Different letters indicate a pairwise comparison with *p* ≤ 0.05.

Females gained significantly more body mass during the two months of the experiment compared to males, regardless of the diet consumed (Figure 3, diet ×sex: F_4,0.097_ = 0.47, *p* = 0.751; sex: F_1,3.385_ = 66.60, *p* < 0.0001; diet: F_4,0.446_ = 2.19, *p* = 0.079).

### 3.2. Females Live Longer Than Males

**Figure 4:**
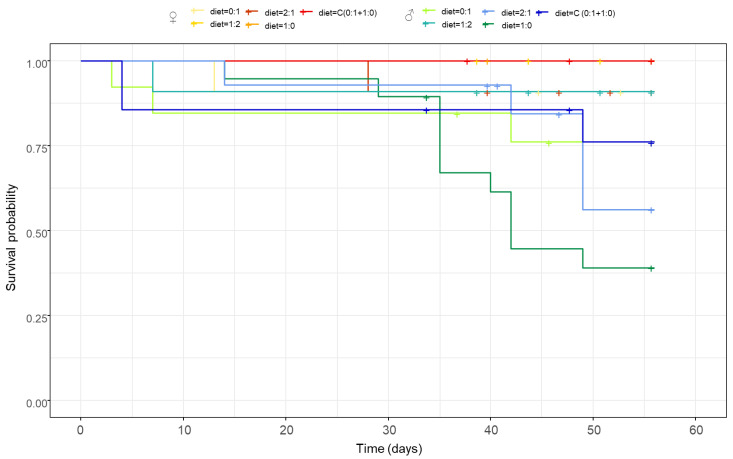
Effect of the P:C ratio of the diet (fixed diets: 0:1, 1:2, 2:1, and 1:0 P:C; and choice diet: C (0:1 + 1:0)) on the survival of female (♀) and male (♂) individuals.

The dietary P:C ratio did not affect survival, and females exhibited higher survival, regardless of the diet consumed (Figure 4, diet × sex: z = −0.99, *p* = 0.321; diet: z = 0.51, *p* = 0.604; sex: z = 1.95, *p* = 0.050).

### 3.3. Only Male Personality Is Affected by the Diet over Time

**Figure 5:**
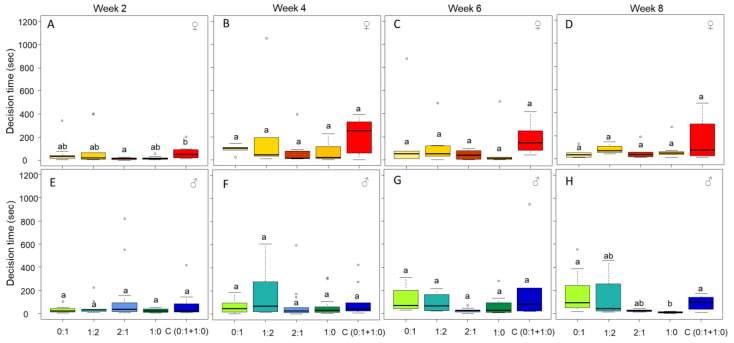
Decision time to leave the shelter per week for females (week 2 (**A**), week 4 (**B**), week 6 (**C**), and week 8 (**D**)) and males (week 2 (**E**), week 4 (**F**), week 6 (**G**), and week 8 (**H**)). Individuals fed on the four different fixed diets (0:1, 1:2, 2:1, and 1:0 P:C) and on the choice diet (C(0:1 + 1:0)). The boxes represent the first and third quartiles and the median. The whiskers represent the maximum and minimum values. The circles represent the outliers. Different letters indicate a pairwise comparison with *p* ≤ 0.05.

The diet influenced the decision time to leave the shelter only at the beginning of the experiment for the females (Figure 5A, week 2: H = 10.88, DF = 4, *p* = 0.027; Figure 5B, week 4: H = 3.62, DF = 4, *p* = 0.458; Figure 5C, week 6: H = 6.17, DF = 4, *p* = 0.186; and Figure 5D, week 8: H = 2.16, DF = 4, *p* = 0.705) and over the weeks for males (Figure 5E, week 2: H = 2.87, DF = 4, *p* = 0.578; Figure 5F, week 4: H = 3.20, DF = 4, *p* = 0.524; Figure 5G, week 6: H = 9.40, DF = 4, *p* = 0.051; and Figure 5H, week 8: H = 14.85, DF = 4, *p* < 0.01).

The decision time to leave the shelter was stable over the weeks for females fed on the same diet (Figure 5, diet 0:1 P:C, H = 4.53, DF = 3, *p* = 0.208; diet 1:2 P:C, H = 2.38, DF = 3, *p* = 0.496; diet 2:1 P:C, H = 4.26, DF = 3, *p* = 0.234; diet 1:0 P:C, H = 6.24, DF = 3, *p* = 0.100; and choice (C(0:1 + 1:0)) diet, H = 3.13, DF = 3, *p* = 0.371). In males, the decision time to leave the shelter was also stable over the weeks, except for males fed on the diet 0:1 and 1:0 P:C (Figure 5, diet 0:1 P:C, H = 8.89, DF = 3, *p* = 0.030; diet 1:2 P:C, H = 2.24, DF = 3, *p* = 0.523; diet 2:1 P:C, H = 1.50, DF = 3, *p* = 0.681; diet 1:0 P:C, H = 7.94, DF = 3, *p* = 0.047; and choice (C (0:1 + 1:0)) diet, H = 3.87, DF = 3, *p* = 0.275). The diet 0:1 P:C induced a slight increase in the decision time to leave the shelter over the week (Figure 5, week 2 versus week 4, z = −1.36, *p* = 0.341; week 2 versus week 6, z = −2.45, *p* = 0.041; week 4 versus week 6, z = −1.20, *p* = 0.275; week 2 versus week 8, z = −2.52, *p* = 0.070; week 4 versus week 8, z = −1.31, *p* = 0.283; and week 6 versus week 8, z = −0.14, *p* = 0.882), while the diet 1:0 P:C induced a slight decrease (Figure 5, week 2 versus week 4, z = −1.32, *p* = 0.278; week 2 versus week 6, z = −0.71, *p* = 0.568; week 4 versus week 6, z = 0.31, *p* = 0.751; week 2 versus week 8, z = 1.69, *p* = 0.181; week 4 versus week 8, z = 2.72, *p* = 0.038; and week 6 versus week 8, z = 2.04, *p* = 0.123).

The decision time to leave the shelter did not significantly differ between males and females over the weeks (week 2, z = 1334.50, *p* = 0.356; week 4, z = 1106.00, *p* = 0.501; week 6, z = 592.50, *p* = 0.891; and week 8, z= 425.50, *p* = 0.368).

### 3.4. Mobility and Exploration Is Affected by Diet (in Males) and Time

**Figure 6:**
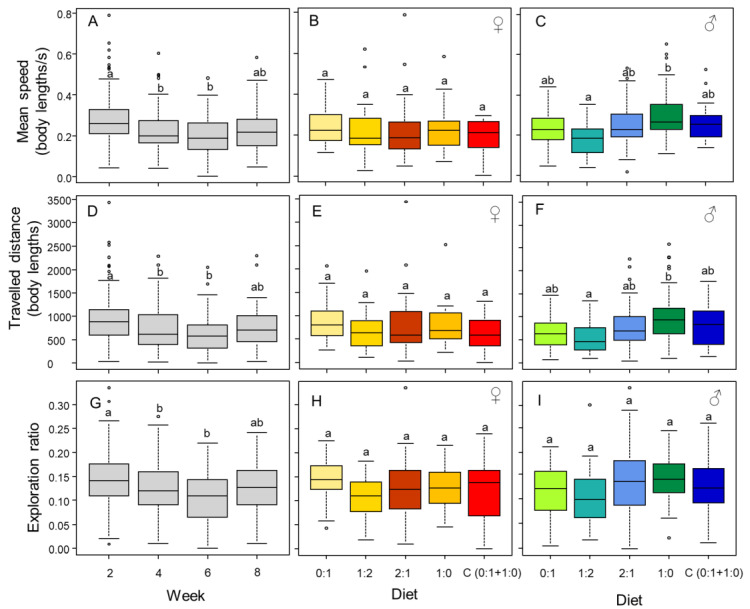
Mobility and exploration. Time affects the speed (**A**), distance traveled (**D**), and exploration ratio (**G**) of individuals. Diet, fixed diet (0:1, 1:2, 2:1, or 1:0 P:C) or choice diet C(0:1 + 1:0)), does not have an effect on these metrics in females (**B**,**E**,**H**), but it does in males (**C**,**F**,**I**). The boxes represent the first and third quartiles and the median. The whiskers represent the maximum and minimum values. The circles represent outliers. Different letters indicate a pairwise comparison with *p* ≤ 0.05.

Individuals tended to be faster (week, F_3,134.69_ = 10.42, *p* < 0.001), traveled more distance (week, F_3,142.11_ = 7.20, *p* < 0.001), and explored more (week, F_3,150.76_ = 8.18, *p* < 0.001) in the first week of observation, although these behaviors tended to increase in the last week of observation (Figure 6A,D,G). Diet differently affected male and female mobility (speed, diet × sex, F_4,104.66_ = 3.31, *p* = 0.013; traveled distance, diet x sex, F_4,103.49_ = 2.70, *p* = 0.034). Female speed and traveled distance were robust across diets (Figure 6B,E,H), but male speed and traveled distance was affected by diet, increasing in the higher P:C ratio diets (Figure 6C,F,I). There was no effect of diet (F_4,1043.26_ = 1.25, *p* = 0.280), sex (F_1,106.68_ = 0.0041, *p* = 0.949), or their interaction (F_4,108.70_ = 0.86, *p* = 0.487) on the exploration ratio. As expected, longer individuals explored more area of the experimental arena (F_1,97.72_ = 6.12, *p* = 0.015).

## 4. Discussion

We found that female cockroaches, unlike males, regulate their intake of protein by consuming less of the diets containing high P:C ratios when restricted to fixed diets and by preferring a carbohydrate-biased diet in the choice experiment. Females also had higher survival and gained more body mass throughout the experiments. After a few weeks of feeding on fixed diets, only male behavior was affected by high P:C ratios. Males became bolder by reducing their decision time to exit the shelter. The analysis of the mobility and exploratory behaviors through video tracking of trajectories also revealed an effect of diet exclusively on males. Additionally, these behaviors were also affected by time, with cockroaches tending to move and explore more at the beginning of the experiment, which might indicate a habituation to the experimental arena. We suggest that the effects observed on survival and behavior only in males might be due to an incapacity to regulate their nutrient intake.

Insects are usually able to regulate their nutrient intake required for growth, development, and reproduction, and nutritional trade-off of life-trait history is commonly observed [30]. In addition, males and females have different nutritional needs, as egg production is more costly than sperm production [36,40,41]. Thus, nutrient intake is supposed to differ between males and females. In the German cockroach *Blattella germanica,* for instance, both sexes prioritize their intake of protein, while females regulate their intake at a 1:2 P:C ratio [42] and males, requiring more dietary carbohydrates, regulate their nutrient intake between a 1:7 and 1:9 P:C ratio, according to their mating history [43]. In the male cockroach *Nauphoeta cinerea*, sperm production is maximized by a P:C ratio of 1:2 [40], while the production of sexual pheromones is optimized by a P:C ratio of 1:8 [44]. However, under dietary choice, both males and females regulate their nutrient intake at a P:C ratio around 1:5, although protein prioritization is more pronounced in females when restricted to a fixed diet [36]. In this study, as seen in other cockroach species [36,42], we found that females of *B. dubia* prioritized their intake of protein, and under dietary choice, in the same manner as females of the German cockroach, they regulated their intake at a 1:2 P:C ratio [42]. Surprisingly, males did not balance their nutrient intake when constrained to fixed diets, and under dietary choice, they selected a high P:C ratio of 1:1.33. Nutrient intake regulation could also be a less important mechanism for males, prioritizing total food intake instead of a given macronutrient. However, the fact that both females and males in the choice diet increased their total food intake by consuming respectively the same quantity of 0:1 and 1:0 diets as when restricted to the fixed diets 0:1 and 1:0 suggests otherwise. Sex differences in the regulation of nutrient intake have also been observed in the cockroach *N. cinerea* [36]. It has been shown that the species *B. dubia* presents a higher content of body protein compared to other cockroach species [45], which could explain the high P:C ratios selected under dietary choice in our study. Female cockroaches exhibit higher fat reserves [46] and consume more food [35], and it is not surprising that females in our experiments ate more than males and gained more body mass.

High P:C ratios are known to decrease survival in many insect species [41,47,48,49,50]. In our study, however, only sex affected the survival of the cockroaches. It has been shown that under starvation, female cockroaches live longer, probably because of their higher fat reserves [46]. Moreover, cockroaches can adjust their behavioral response to extreme nutritional deficiencies by quickly balancing their diets [35]. Since, in our experiments, males did not balance their nutrient intake, their mortality was much higher compared to females.

Maternal diets can influence the personality of the offspring [51], and the diet consumed at the juvenile stage can affect the personality of adults [28,29,33]. Our study is the first to show that the diet consumed at adult stages can affect personality. Only one other study investigated the effect of the P:C ratio on the adult stage and it did not find any personality difference between the diets [33]. However, in that experiment, the authors fed adult crickets with fixed diets for only four weeks before testing their behavior [33]. In our experiments, a slight effect of the diet on the behavior was observed in females at the very beginning of the experiment, but this effect was not consistent over time. In males, we started to observe the effects of the diet only after six weeks of ad libitum feeding. Our results suggest a long-term effect of the diets on personality and indicate the necessity of long-term studies for this research theme.

Sex-specific differences are common in animal personalities [51,52,53,54], including in cockroaches [2]. Han and Dingemanse found that juvenile crickets raised on high P:C ratios exhibited a more aggressive personality once they became adults, but this effect was only present in males, suggesting that males are more sensitive to nutritional stress [33]. Our results also show a sex-specific effect of diet on personality, with high P:C ratios inducing a bolder personality (decrease of the decision time to exit the shelter) in males only. This difference in behavior between males and females could be explained by the fact that males were unable to regulate their nutrient intake; thus, males were incapable of dealing with the effects of high P:C ratio diets, affecting both their survival and behavior. It is important to note that food preferences and intake target can vary from one population to another and from field to laboratory conditions [55]. While our sample size was not large, we had enough statistical power to observe relevant changes in behavior; future work with a larger sample size of individuals from different populations is required to better understand this mechanism.

Bolder individuals take more risks in foraging and therefore have higher probability to find food resources [8]. Social facilitation in feeding, including finding new food resources for the group, is common in gregarious cockroaches [56]. Sex-specific effects of diet on personality in the Dubia cockroach could improve the fitness of the group by allowing males to discover new food resources when facing nutritional stress, while the ovoviviparous females, and the offspring they carry, spend more time protected in the shelter.

## Figures and Tables

**Table 1:** Percentage by mass of the various ingredients added in the four artificial diets.

P:C Ratio	Amino Acids *	Sucrose	Cholesterol	Wesson’s Salt	Vitamin Mix
0:1	0	96.45	0.55	2.50	0.50
1:2	32.15	64.3	0.55	2.50	0.50
2:1	64.3	32.15	0.55	2.50	0.50
1:0	96.45	0	0.55	2.50	0.50

* The following amino acids were used in equal quantity in all diets: alanine, arginine, aspartic acid, asparagine, cysteine, glutamic acid, glutamine, glycine, histidine, isoleucine, leucine, lysine, methionine, phenylalanine, proline, serine, threonine, tryptophan, tyrosine, and valine.

## Data Availability

The raw data are available from the corresponding author upon request.
